# Competing Conservation Objectives for Predators and Prey: Estimating Killer Whale Prey Requirements for Chinook Salmon

**DOI:** 10.1371/journal.pone.0026738

**Published:** 2011-11-09

**Authors:** Rob Williams, Martin Krkošek, Erin Ashe, Trevor A. Branch, Steve Clark, Philip S. Hammond, Erich Hoyt, Dawn P. Noren, David Rosen, Arliss Winship

**Affiliations:** 1 University of Washington, Seattle, Washington, United States of America; 2 Marine Mammal Research Unit, University of British Columbia, Vancouver, Canada; 3 Department of Zoology, University of Otago, Dunedin, New Zealand; 4 Oceans Initiative, Pearse Island, British Columbia, Canada; 5 School of Aquatic and Fishery Sciences, University of Washington, Seattle, Washington, United States of America; 6 SeaWorld, Orlando, Florida, United States of America; 7 Sea Mammal Research Unit, University of St. Andrews, St. Andrews, Scotland, United Kingdom; 8 Whale and Dolphin Conservation Society, North Berwick, Scotland, United Kingdom; 9 National Marine Fisheries Service, Northwest Fisheries Science Center, Marine Mammal & Seabird Program, National Oceanic and Atmospheric Administration, Seattle, Washington, United States of America; 10 Marine Mammal Research Unit, Fisheries Centre, Department of Zoology, University of British Columbia, Vancouver, British Columbia, Canada; 11 Life Sciences Centre, Department of Biology, Dalhousie University, Halifax, Nova Scotia, Canada; University of British Columbia, Canada

## Abstract

Ecosystem-based management (EBM) of marine resources attempts to conserve interacting species. In contrast to single-species fisheries management, EBM aims to identify and resolve conflicting objectives for different species. Such a conflict may be emerging in the northeastern Pacific for southern resident killer whales (*Orcinus orca*) and their primary prey, Chinook salmon (*Oncorhynchus tshawytscha*). Both species have at-risk conservation status and transboundary (Canada–US) ranges. We modeled individual killer whale prey requirements from feeding and growth records of captive killer whales and morphometric data from historic live-capture fishery and whaling records worldwide. The models, combined with caloric value of salmon, and demographic and diet data for wild killer whales, allow us to predict salmon quantities needed to maintain and recover this killer whale population, which numbered 87 individuals in 2009. Our analyses provide new information on cost of lactation and new parameter estimates for other killer whale populations globally. Prey requirements of southern resident killer whales are difficult to reconcile with fisheries and conservation objectives for Chinook salmon, because the number of fish required is large relative to annual returns and fishery catches. For instance, a U.S. recovery goal (2.3% annual population growth of killer whales over 28 years) implies a 75% increase in energetic requirements. Reducing salmon fisheries may serve as a temporary mitigation measure to allow time for management actions to improve salmon productivity to take effect. As ecosystem-based fishery management becomes more prevalent, trade-offs between conservation objectives for predators and prey will become increasingly necessary. Our approach offers scenarios to compare relative influence of various sources of uncertainty on the resulting consumption estimates to prioritise future research efforts, and a general approach for assessing the extent of conflict between conservation objectives for threatened or protected wildlife where the interaction between affected species can be quantified.

## Introduction

Ecosystem-based management (EBM) of marine resources is widely recognized as the next step in achieving conservation and fishery objectives, while benefiting from lessons learned from the successes and failures of single-species fisheries management [1]–[3]. EBM includes incorporation of species interactions when setting conservation and fishery objectives [2], [4]. EBM approaches are articulated as part of marine policy in many countries [5]–[7], although implementation is farther along in some regions than others. For example, predator requirements are formally considered in the management of Antarctic krill fisheries off South Georgia, such that body condition of krill predators can be used as an indicator to trigger reduction in fishery quotas [8]. For North Sea cod (*Gadus morhua*), the International Council for the Exploration of the Seas (ICES) has incorporated estimates of grey seal prey requirements to ensure that this predator is considered when setting fisheries quotas [9]. A related management tool used by ICES is the Ecological Quality Objective, an example of which is that changes in sandeel (*Ammodytes marinus*) fishery management actions are triggered if breeding success of black-legged kittiwakes (*Rissa tridactyla*) falls below a predetermined threshold [9]. Hence, an EBM approach could involve an explicit allocation of fishery quota for ecosystem or predator needs [10], [11].

Oceans policies in Canada and the United States are shifting toward EBM [12], [13], but implementation requires that practical choices be made, particularly when there are conflicting objectives for different species [14]. In fact, a key requirement of EBM is identification of conflicting objectives, so that trade-offs can be proposed to achieve multiple objectives [15]. A particularly challenging case occurs when two species are endangered, but one eats the other [16]. Such a situation is emerging for southern resident killer whales (‘SRKW’, *Orcinus orca*) and Chinook salmon (*Oncorhynchus tshawytscha*) in the Salish Sea, bordered by northern Washington State (WA), USA and southern British Columbia (BC), Canada.

Unlike the mammal-eating transient killer whale population, SRKWs prey exclusively on fish, and specialize almost exclusively on Chinook salmon [17], [18]. SRKWs have been assessed as one of the most critically endangered marine mammal populations in US waters [19]. Many of the salmon runs on which this predator depends are also depleted, although not all depleted salmon stocks have been formally listed as endangered [20]–[22]. In Canada, stocks of commercially valuable marine fish species are normally managed using traditional fisheries-management tools, rather than endangered species legislation [23]. By 1998, the total Canadian catch of all salmon species was at its historic low for the 20^th^ Century, with Chinook and coho experiencing the most severe declines [24]. Historical (late 1800s to early 1900s) annual run size of Chinook salmon to the Fraser River system was nearly 1 million [25]. However, BC salmon stocks in general are estimated to be at 36% of historical (1800s) run size, and Puget Sound stocks at 8% [26]. A number of scientific and socio-economic factors are considered when deciding whether to list depleted populations of exploited species under endangered species legislation. On the other hand, many species have not been formally evaluated. Consequently, the paucity of exploited species on a country's endangered species lists does not necessarily indicate favourable conservation status [23].

There is a strong ecological link between killer whales and salmon, with both circumstantial and direct evidence for prey selectivity in resident killer whales [17], [18], [27]. Critical group size of foraging ‘northern resident’ killer whales (off northern Vancouver Island) was correlated with inter-annual variability in Chinook salmon abundance, but not with the abundance of four other salmon species in the region [28]. Dedicated field studies have demonstrated prey selectivity, in that resident killer whales were found to target Chinook salmon even when Chinook abundance was low relative to other salmonids [17]. In fact, it has been suggested that resident killer whales are “highly specialized and dependent on this single salmonid species to an extent that it is a limiting factor in their population dynamics” [29]. As a result of this specialization, reduced availability of Chinook salmon is linked to increased adult mortality [29] and reduced reproduction [30] of resident killer whales.

One stated U.S. recovery goal for southern resident killer whales is an average annual growth of 2.3% over 28 consecutive years [31], which is expected to increase predation pressure on Chinook salmon stocks. The conservation and management objectives for salmon in this transboundary region are more difficult to sum up in one sentence. The Pacific Salmon Commission is an advisory body formed by the Governments of Canada and the United States to implement the Pacific Salmon Treaty. The Commission aims to offer advice that achieves two broad management objectives, (http://www.psc.org/about_role.htm): “first, to conserve the Pacific Salmon in order to achieve optimum production, and second, to divide the harvests so that each country reaps the benefits of its investment in salmon management.”

Populations of both killer whales and Chinook salmon are in need of rebuilding. However, there is strong potential for these objectives to conflict, given the nearly exclusive dietary specialization on Chinook salmon by the former. Good estimates of prey (energetic) requirements of killer whales are needed to evaluate the extent to which management objectives for these predators and their prey are in conflict, and to offer advice for resolving conflict. Previous attempts to estimate killer whale prey requirements have used metabolic rates measured from two captive animals of unknown mass [32] or estimated field metabolic rates from daily activity budgets of wild whales, scaled up to population-level requirements [33]–[35]. Drawing inferences from such data is clearly unsatisfactory, but there is little information available about how mass-specific metabolic rates of killer whales depend on sex, reproductive status, age, and size [34].

Conflicts between species occur in many places, but management actions to mitigate conflicts can have important and potentially undesirable consequences [36]. For example, grey seal culls to protect breeding burrows of puffins on the Farne Islands, UK led to the establishment of new seal colonies and a substantial increase in grey seal population growth [37], increasing conflict with fisheries in the North Sea [38]. More recently, the establishment of Special Areas for Conservation under the EU Habitats Directive for harbour seals and Atlantic salmon in Scotland raises issues similar to those for SRKWs and Chinook salmon [39]. Notwithstanding the difficulties involved in predicting ecosystem response to management action, any evaluation of the potential for conflict between fisheries and marine mammals will involve generating good estimates of prey required to sustain and recover the predator population [40].

Using data from several sources, we modelled morphometrics and energetics of killer whales of different age-sex classes. Our analyses draw on extensive records from several killer whale ecotypes and geographic regions based on historic live-capture fisheries and whaling records. The results thereby also provide new parameter estimates that can be used globally in marine ecosystem models that include killer whales. We applied the model to the known sex, size, and age distribution of the 87 individuals in the SRKW population in 2009 (courtesy of Center for Whale Research, Friday Harbor, WA) to estimate the energetic needs and corresponding number of Chinook salmon required for maintenance and recovery of the endangered SRKW population. Finally, we compare the estimates of SRKW consumption of Chinook with fisheries stock assessment data for Chinook salmon from the Fraser River to demonstrate the potential for conflict between objectives for killer whale conservation and salmon fisheries management. Our analyses therefore serve a dual mandate: to provide new parameter estimates for ecosystem models that include killer whales around the world; and to illustrate one way that the approach could be used, using southern resident killer whales as a data-rich case study.

## Methods

We developed a set of statistical models for the relationships among age, length, mass, sex, and energy consumption of captured and captive killer whales and then applied the models to the known age and sex distribution of the SRKW population. An important assumption of our approach is that the statistical relationships we estimated for killer whales are transferable among live-capture, captive, and wild populations. We therefore compared results from models from these different sources of data as a check, where comparable data were available.

The models yielded an estimate of the gross energy requirements of the SRKW population, which we converted to Chinook salmon consumption based on the average energy content of an individual adult Chinook salmon. The estimated salmon requirement of the SRKW population was then compared to fisheries data of catch and escapement of Chinook salmon populations upon which the SRKW population feeds. Full details of the modeling approach are given in the supporting information (Text S1). Below we provide a cursory overview.

First, we modeled length at age from time-series data of 30 captive killer whales (29 of Icelandic origin and one female northeastern Pacific northern resident ecotype). We fitted and compared three growth models – von Bertalanffy, Gompertz, and logistic growth [41] – using a nonlinear mixed-effects modeling approach to account for the repeated measures structure of the data [42]. The model allowed for differences in growth rates and asymptotic length between male and female whales, thereby capturing the relationships among sex, age, and length. We checked the estimated asymptotic lengths of the models fit to the captive whale data against global capture records from the International Whaling Commission and other sources (Text S1).

Next, to check correspondence of allometric relationships between wild and captive animals, we modeled mass-at-length from live-capture records [43] as well as that for captive killer whales held by SeaWorld according to 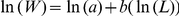, where W is mass in kg and L is length in cm. For live-capture records, we compared models with mixed and fixed effects on ln(*a*) and *b* to accommodate possible variation among four ecotypes: Icelandic (n = 11); northern resident (n = 15); southern resident (n = 18); and North Pacific mammal-eating or *transient* killer whales (n = 4). For the captive killer whales, we included a random effect on the intercept and slope to account for the repeated measures structure of the data [42].

To model gross energy requirements of killer whales, we used data on food consumption from captive animals held by SeaWorld (Text S1; Table S1) according to 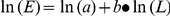 where *E* is the energy consumed per day in kcal and *L* is the length of the whale in cm. We modeled males and females separately, and further placed females into one of four reproductive categories: (1) ‘Single’ (neither pregnant, lactating, nor immediately post-lactation); (2) ‘Pregnant’ (inferred from blood hormone levels); (3) ‘Lactating’ (recorded until month 3 post-birth); and (4) ‘Post-lactation’ (six months after lactation records end to allow for possibility of continued partial lactation and a recovery period). The sex and reproductive categories were included as fixed effects in the model and random effects were further included to account for the repeated measures structure of the data [42].

Finally, we applied the above models to the 2009 SRKW population (i.e., 87 individuals) using data on age, sex, and reproductive status as inputs to predict the lengths, weights, and energy needs of SRKWs (demographic data used with permission from Ken Balcomb, Center for Whale Research; see Text S1). Because of the high degree of prey-selectivity of SRKWs on Chinook salmon, we estimated the amount of Chinook salmon required to meet the energetic needs of the SRKW population in 2009. We therefore converted the estimated caloric demand to units of fish, in two ways. First, we considered a value (16,386 kcal/fish) of a Chinook salmon of unknown size sampled in the Salish Sea summer core habitat for SRKWs [34]. Secondly, we placed upper and lower bounds on this measure, by estimating the caloric value of the SRKW's preferred prey item (a 4-year-old Chinook salmon of length 81 cm and mass 8.5 kg; [17]) using two previously reported estimates of energy density: a “calorie-rich” estimate (18,700 kcal per fish; [44, p. 57]), and a “lean” or energy-sparse estimate (10,869 kcal/fish; [45]). Because our model was based on gross energy consumption from different prey types, our conversion to Chinook biomass assumes a reasonably equivalent transformation from gross to net energy in wild and captive killer whales (i.e., ∼85% [32]). We then compared this estimate of required biomass with escapement and catch data from Chinook salmon populations upon which the SRKWs feed.

From diet studies conducted in summer months near the San Juan Islands, it is estimated that 83% of the SRKW diet is composed of Chinook salmon, 90% of which were of Fraser River origin [18]. One study reported that SRKWs were found within inland waters near the San Juan Islands approximately 79% of days from May–September [46]. We scaled our plausible summertime estimate of Fraser River Chinook consumption accordingly ( = total consumption * 0.83 * 0. 90 * 0.79). Availability of Fraser River Chinook in the marine environment is approximated by the sum of: (a) the number of fish taken by the whales; (b) takes by commercial and recreational fisheries, which averaged 18,000 annually from 2004–2008 in the Strait of Georgia [47]; and (c) terminal run size (number of fish available after marine predation and at-sea mortality), which is approximately 300,000 annually [47]. Note that although in-river fisheries by sport and First Nations fisheries increase average annual fishery takes to 64,000 [47], in-river fisheries remove fish after they have been counted in the terminal run size, and are therefore excluded from these calculations.

## Results

**Figure 1:**
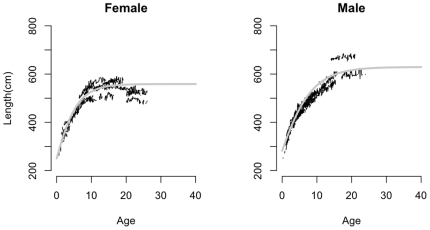
Length at age plots (dots represent each monthly measurement), with model predictions (grey line) for male and female captive whales from SeaWorld records. Males achieve a greater asymptotic length than females.

The best-fit growth model (i.e., length-at-age model) for captive killer whales was the Gompertz model (Text S1; Table S2), which agreed well with the data (Figure 1). Only weak statistical evidence was found to support differences in growth parameters between males and females (ΔAIC = 1.08; log-likelihood ratio test P = 0.079). However, because males reach a larger size than females [48], [49] we included sex as a fixed factor in the model for the parameter estimation (Text S1). The estimated asymptotic lengths of killer whales were smaller than those for the SRKW population estimated from catch records (Text S1). We therefore considered various scenarios for the model predictions based on a range of plausible values for asymptotic length of SRKWs.

**Figure 2:**
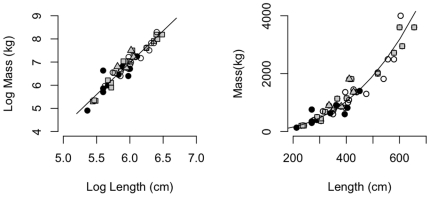
Mass at length on a log (left plot) and linear (right plot) scale for each of four killer whale ecotypes. Icelandic killer whales (black circles); northeast Pacific “northern resident” killer whales (grey squares); northeast Pacific “southern resident” killer whales (open circle); and northeast Pacific “transient” killer whales (triangles) from historic live-capture fisheries.

The statistical model for mass-at-length of captive and live-capture killer whales agreed well with the data (Figure 2). The data did not support inclusion of random effects or fixed effects for ecotype, so we pooled the data for parameter estimation. Parameter estimates from live-capture and captive animals for the mass-at-length model were very similar (live-capture: *a* = 6.7e-05, 95% CI: 1.7e-05 to 2.6e-04 and *b* = 2.8, 95% CI: 2.5 to 3.0; captive animals: *a* = −9.4, 95% CI: −9.2 to −9.6 and *b* = 2.73, 95% CI: 2.69 to 2.76; Text S1).

**Figure 3:**
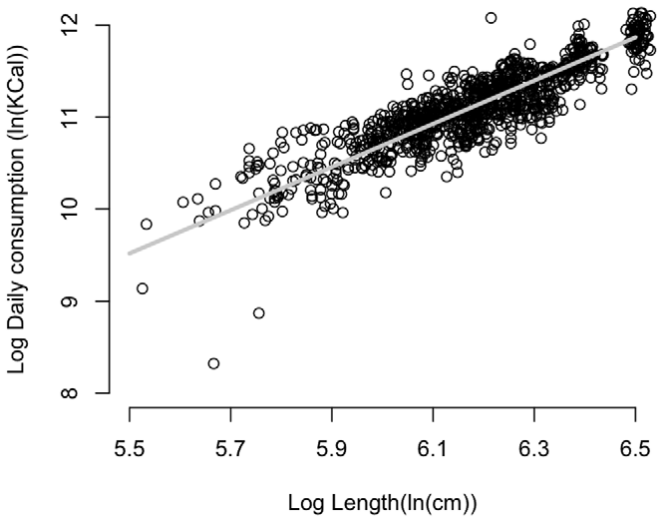
Estimated daily energy consumption (Kcal) at length (cm) on natural log scale, predicted from captive male killer whale records. Parameters are −3.4 for the intercept and 2.35 for the slope.

The statistical model for daily energy consumption of captive killer whales agreed well with the data (Figure 3). There was strong support for a model in which the intercept varied according to reproductive class, which indicated that lactation is associated with a large increase in energy consumption (ΔAIC = 46.92; log-likelihood ratio test P<0.001; Text S1). For instance, prey intake for a 32-year-old female during lactation was 42% higher than when not lactating (Table S3). Parameters of the fixed effects in the energy model and the 95% confidence intervals are provided in the Supplementary Information (Text S1; Table S4).

By applying the length-at-age and energy consumption models to the age and sex data for the SRKW population, we calculated annual Chinook consumption estimates for the SRKW population (Table 1). However, these estimates were affected by three main sources of uncertainty: assumed asymptotic length in the wild killer whale population; proportion of Chinook in the whales' diet; and sparse information on winter diet. We determined that the 80^th^ percentile of the length distribution in North Pacific whaling records was the best estimate of asymptotic body length (Table 1; see explanation in Text S1, and body length estimates for killer whales from other geographic regions in Table S5). These values are nearly identical to the asymptotic lengths of male and female southern resident killer whales determined by photogrammetric methods [62]. If SRKWs met their energetic requirements exclusively through Chinook salmon (i.e., the “100% scenario”), the 2009 SRKW population would require the energetic equivalent of approximately 241,500 Chinook salmon annually (Table 1). Given the best estimate of body size, but allowing for uncertainty in the caloric value of Chinook salmon, we find that this annual requirement could be as low as 211,600 or as high as 364,100 (Table 2).

**Table 1:** Daily and annual energetic requirements in kcal and number of Chinook salmon (based on a hypothetical 16,386 kcal salmon, [34]) for the current size of the southern resident killer whale (SRKW) population, considering various levels of asymptotic length and mass attained by killer whales in the population.

Body Length	Male		Female		Total Energy Requirement		Total Chinook Requirement		
Scenario	Asymptotic		Asymptotic		kcal	kcal	“100%”	“100%”	“Summer”
(Source)	Length (cm)	Mass (kg)	Length (cm)	Mass (kg)	(×10^6^) (per day)	(×10^9^) (per year)	fish (per day)	fish ×10^3^ (per year)	Fish ×10^3^ (per year)
IWC NP Max	820	9655	780	8393	17.8	6.5	1088	397	98
IWC NP 99^th^	804	9137	742	7298	16.2	5.9	988	361	89
IWC NP 95th	770	8096	710	6451	14.4	5.3	880	321	79
**IWC NP 80^th^**	**700**	**6199**	**630**	**4616**	**10.8**	**4.0**	**662**	**242**	**59**
SeaWorld Max	685	5835	626	4534	10.5	3.8	639	233	57
SeaWorld 99^th^	678	5669	620	4413	10. 2	3.7	622	227	56
SeaWorld 95^th^	651	5059	598	3989	9.2	3.3	562	205	50
SeaWorld 80^th^	604	4102	560	3319	7.7	2.8	468	171	42

Our best estimate of body size in SRKW is based on the 80^th^ percentile of body lengths from the IWC catch records from the North Pacific, shown in **bold**. The “100%” scenario is hypothetical and illustrative: it naively converts caloric requirement to units of fish, assuming that the diet is composed entirely of Chinook salmon. The “Summer” scenario only estimates prey requirements from May–September, based on the proportion (83%) of the diet that is estimated to come from Chinook salmon in summer [17], [18]. Note that a ‘recovered’ population refers here to 155 animals in 2029 (one scenario calculated from the 2001 population of 81 animals with an estimated average annual growth of 2.3 percent over the succeeding 28 years, [31]). A recovered population will require at least 75% more energy than the values predicted here.

**Table 2:** Estimated prey requirements of wild killer whales, based on two plausible values for calorie content of a typical, 4-year-old Chinook salmon.

Body Length	“Calorie-rich” Chinook Scenario			“Lean” Chinook Scenario		
Scenario	“100%”	“100%”	“Summer”	“100%”	“100%”	“Summer”
(Source)	fish (per day)	fish ×10^3^ (per year)	fish ×10^3^ (per year)	fish (per day)	fish ×10^3^ (per year)	fish ×10^3^ (per year)
IWC NP Max	953	348	86	1640	599	147
IWC NP 99^th^	866	316	78	1489	544	134
IWC NP 95th	771	282	69	1327	484	119
**IWC NP 80^th^**	**580**	**212**	**52**	**998**	**364**	**90**
SeaWorld Max	559	204	50	963	351	86
SeaWorld 99^th^	545	199	49	938	342	84
SeaWorld 95^th^	493	180	44	848	309	76
SeaWorld 80^th^	410	150	37	705	257	63

Salmon of length 81 cm and mass 8.5 kg, the preferred size of Chinook prey of the resident killer whales [17]. Energy requirements predicted for southern resident killer whale (SRKW) population assume that activity levels are equal in captivity and in the wild. The “calorie-rich” scenario assumes an average energy density of 2,200 kcal/kg [44, p. 57)]; therefore each Chinook was estimated to represent 18,700 kcal. The “lean Chinook” scenario uses the mean of 5 Chinook of unknown size, collected in the Gulf of Alaska (mean = 5.35 kJ/g = 10,869 kcal/8.5 kg fish; [45]). Note that the row in bold type represents the most plausible estimate, based on observed body sizes from whaling records from the northeastern Pacific. The SRKW population in 2009 consisted of 87 individuals of the following age-sex classes (Center for Whale Research): females with calves (10); adult males (23); juveniles (15); calves (10); and adult females without calves (29).

Clearly, the killer whale diet is not composed entirely of Chinook, so our “summer” estimate represents a more plausible summary of the various studies published to date. In summer months (May through September), 83% of the SRKW diet is composed of Chinook salmon, 90% of which are of Fraser River origin [18]. SRKWs are found in their core summer habitat on 79% of days from May–September [46]. We present plausible estimates of SRKW pressure on Fraser River Chinook stocks (“Summer” estimates in Table 1) by multiplying the annual energetic demand by 24.6% (namely 5/12 * 0.83 * 0.90 * 0.79). Depending on body size, the best estimate of the summer demand on Fraser River Chinook is 59,384 (range 42,000–97,600; Table 1). Depending on caloric value of Chinook and given the best estimate of body size, these lower, “summer” estimates may be as low as 52,000 and as high as 89,500 (Table 2) Fraser River Chinook consumed annually by SRKWs in core habitat in summer months.

The above calculations can be used to estimate the proportion of the Fraser River Chinook salmon population that is consumed annually by SRKWs (see Methods). Average fisheries catch of Fraser River Chinook is 18,000 fish and average terminal run size is 300,000 [47]. These numbers suggest that SRKWs may consume 12% (42,000/(42,000+18,000+300,000)) to 23% (97,600/(97,600+18,000+300,000)) of available Fraser River Chinook in the region from May–September. These estimates only include information available for the whales' core summertime habitat. Total abundance of Chinook may be higher than our estimates suggest because, north of the Salish Sea, Chinook salmon are harvested in commercial and sport fisheries, and by-caught in fisheries for other salmon. On the other hand, SRKWs spend little time there. We focus on the Salish Sea, because considering Chinook salmon availability in northern waters would require us also to consider the substantial demands for northern resident killer whales of Chinook salmon in that habitat. Our analyses suggest that takes of Fraser River Chinook by SRKWs may well exceed those from all fisheries in the Salish Sea. If the SRKW population reached 155 animals by 2029 (one recovery scenario reported in [31]), energetic requirements could become ∼75% higher than those reported here.

## Discussion

The number of Chinook salmon required to maintain the endangered southern resident killer whale population at its current size is substantial, and large enough to warrant explicit treatment in endangered species recovery [50] and fisheries management [40] plans. The SRKW population, numbering only 87 individuals in 2009, may easily consume 12–23% of available Fraser River Chinook in the region from May–September. These plausible summer estimates are large relative to the 10–40% natural mortality considered in regional fisheries stock assessment models [51], [52], because they ignore likely consumption of Chinook in winter months, consumption by parapatric northern resident killer whales, consumption by Steller sea lions, sharks and other predators [53], [54], and the fact that wild, free-ranging killer whales likely have higher metabolic demands than captive animals. Consumption of Fraser River Chinook by SRKWs may exceed those from all fisheries in the region combined. Prey requirements of a “recovered” population could be ∼75% higher than those reported here, but it is unclear when along the recovery process management needs to shift to account for the demands of this larger population. The plausible summertime estimates we present build a compelling case for competition between conservation objectives for killer whales and Chinook salmon, even at the killer whale population's current size.

One criticism of efforts to switch to EBM is a perception that the data requirements are simply too onerous [1]. Here we show that in the face of such complexity, it makes sense to start with the most data-rich component, or the predator-prey interaction suspected to account for the greatest source of predation. By starting with one simple component, it is possible to evaluate whether that interaction alone suggests competition. Demonstrating competition is in itself not sufficient for management action. One needs quantitative results that show that the likely impact of the scenarios modelled requires action in order to meet objectives, which is the case in our example. If that can be shown with a simple model that includes only minimum-case scenarios, there is sufficient evidence to warrant some management action. More complex representations of reality can always be built up over time (e.g., adding sharks and other fish, other marine mammals etc) as needed. In our example, the point estimates of prey requirements emerging from various scenarios we considered collectively span an order of magnitude. But even the lowest estimates produced from the scenarios we considered indicate killer whale prey requirements that are large relative to availability and fisheries removals, and suggest that the ecosystem as it stands has limited capacity to meet both needs.

Our predictions of prey requirements of individual killer whales and their populations are presented as a range of values under different scenarios, rather than as a single probability distribution. In part, this is intended to maintain sufficient transparency about the various model components to allow the various point estimates to be used in ecosystem models that include killer whales with different diets living in other parts of the world. To inform conservation and management of our particular population of interest, southern resident killer whales, we have begun to quantify levels of uncertainty arising from a few key sources, namely killer whale body length and caloric value of Chinook. In practice, it is only the abundance of killer whales in the population that is known with certainty in this case. Some parameters we are treating as constants, namely proportion of time spent in the core summer range and proportion of Chinook in the summer diet are in fact estimates with associated levels of uncertainty. These sources of uncertainty were ignored in our calculations to illustrate potential magnitude of conflict, but when scaled up to the population level, could be substantial. For example, we considered that killer whales were found in their core summertime habitat on 79% of days from May–September [46], but more recent analyses using additional data [55] suggest that this parameter may now be revised downward to 53% with a CV of 5% (i.e., 95% CIs: 49–58%). Similarly, as killer whale diet studies progress and the sample sizes increase, a priority will be to put confidence limits on the proportion of diet that is composed of Chinook salmon, and the proportion of Chinook from the Fraser River [18]. Ultimately, all of these sources of uncertainty need to be combined into a single analytical framework that can provide the best possible estimate of uncertainty. An obvious next step is to conduct a sensitivity analysis to identify which parameters have the strongest impact on model results, to prioritise future research efforts to reduce sources of uncertainty where feasible. Ideally, one would thoroughly integrate all possible sources of uncertainty in a simulation framework (i.e., one that allows all model parameters to vary randomly within our best estimate of sample distributions over large numbers of iterations), but this is beyond the scope of the current study.

Our conclusion that the competition will increase as killer whale populations recover presupposes that killer whales will not respond to scarcity by switching prey. In contrast to generalist predators [56], the decades-long studies of resident killer whales show that hunting specialization and prey selectivity constrain the whales' ability to switch prey in times of reduced prey availability to the degree that adult survivorship and reproduction are reduced [16], [29], [30]. Of course, existing data span only moderate ranges of Chinook abundance [e.g., 28]. We do not know how the predator, prey or other components of this ecosystem would behave at extremely high or low prey densities. We account only for direct, numerical effects, and have limited ability to forecast how the system would respond to increased nutritional demands of a recovered population of killer whales. In other words, we can quantify how the caloric demands of a recovered killer whale population would increase, but are limited in our ability to predict how, ecologically, predators might meet those demands. Although it is well established in other ecological systems that there can be behavioural responses of predator and/or prey to situations where a preferred prey is at low availability, for killer whales and Chinook salmon, we simply do not know. Future research efforts should consider trait-mediated or indirect effects [57], [58], because the net effect of these food-web interactions could increase or decrease net impact of killer whales on Chinook, depending, for example, on food web topology and various interaction strengths, behavioural responses, functional responses and density dependence.

Our predictions of the energy requirements of individual SRKWs based on prey intake of captive animals and body lengths of wild whales agree reasonably well with previous estimates [33], [34], [59]. More importantly, our estimates offer parameter estimates across a range of scenarios that can be used to guide future research and pose testable hypotheses about likely outcomes of different fishery management actions. As an example of the former, our predictions vary by a factor of 2.3, depending on the value used for body size (Table 1). Uncertainty in body size should be incorporated in ecosystem models, especially given recent evidence for multiple killer whale species worldwide [60]. Laser-metric and photogrammetric methods [61], [62] can be used to measure length of free-ranging killer whales of any population of interest, and this would be a better metric to use in an ecosystem model than the length of a generic killer whale. Our predictions vary by a factor of 2.9, depending on whether all or none of the SRKW *winter* diet is composed of Chinook salmon. The predictions vary by a factor of 1.7, depending on the caloric value we use for a typical Chinook. For logistical reasons, body size and caloric value of prey are much more tractable problems to solve than winter diet studies, so these research items should be added to the agenda to improve the precision and accuracy of estimates. Our framework can estimate how much prey is required to support a predator population, but our estimates make untested assumptions about foraging efficiency of killer whales that merit additional, dedicated field research.

The US National Marine Fisheries Service does consider SRKW prey requirements in fisheries management decisions and report these in the biological opinions they are required to produce under the US Endangered Species Act [e.g., 55]. However, current fisheries models in the region are not equipped to anticipate the needs of a growing killer whale population because they are designed primarily for hindcasting, rather than forecasting [51], [52], [63]. A multi-species or ecosystem approach to fisheries stock assessments and management may be needed to balance the needs of the fishery (First Nations, North American Tribes, commercial and recreational) and the needs of the endangered species (to maintain killer whales at their current population size and to promote recovery). This would raise new policy issues to evaluate how the prey requirements of a transboundary species might be considered in relation to existing bilateral quota allocations under the Pacific Salmon Treaty. An intriguing policy solution would be to give killer whales a salmon catch allocation under the Treaty. This would be consistent with the spirit of Canada's Wild Salmon Policy, which places conservation needs ahead of fishery allocations.

Information on prey requirements of top predators can be used to inform models to improve management of both predator and prey, but the complexity of the dynamic systems being modelled will require us to provide scientific advice iteratively [64]. A pragmatic approach that acknowledges ecological complexity while favouring simplistic models may be preferred in cases where scientific advice must be provided on time-sensitive issues [65]. Chinook stock assessment models currently assume that if natural mortality (i.e., predation) increased, there would be a compensatory, density-dependent response in which Chinook mature earlier [47], [52]. This assumption warrants additional investigation. If compensatory density-dependent decreases in age at maturity or increases in survival [66] are insufficient to compensate for high predation rates, then stocks may take longer to recover than basic models assume.

The complexity of the next steps is non-trivial. Providing scientific advice to inform management often runs into a recurring suite of problems: scaling up from metabolic rates of individuals invariably involves some degree of extrapolation beyond the range of available data; model predictions are difficult to ground-truth with direct observations of prey intake; and/or incorporation of all sources of uncertainty result in such broad confidence intervals as to render the resulting parameter estimates of limited practical value [34], [67]. Our case study is a useful way to explore these issues, because resident killer whale diet is comparatively simple, and demographic data represent the upper limit of what is likely to be available for any cetacean population [29]. Our analyses illustrate the point that little ecosystem complexity need be considered before the limitations of a single-stock fisheries management plan become apparent [68].

The dilemma is this: if conservation objectives for killer whale population recovery are actually achieved, the resulting increase in predation pressure on Chinook stocks will compromise the ability to meet recovery objectives for Chinook salmon. In light of competing objectives, a precautionary management strategy would reduce fisheries quotas temporarily while implementing actions that encourage greater production of Chinook salmon. Actions to encourage production may include removing dams, restoring spawning habitat, providing safe passage to out-migrating smolts and disease control of fish farms [69], [70]. Hatchery production could be an option, but it remains to be seen whether hatcheries “may cause more harm than good” [71]. Our point is that history indicates that there is ample scope for increasing returns of wild Chinook salmon to the Fraser River well above current annual averages of 300,000. In 1908, an estimated 690,000 Chinook salmon returned to Puget Sound alone [71]. Temporary reductions in fisheries quotas may buy some time while salmon spawning habitat is improved to increase salmon returns [69]. These actions may go a long way toward mitigating conflict between recovery of both predator and prey.

When one protected species relies almost exclusively on another protected species, it can be difficult to develop management frameworks that meet the needs of both species. This can lead to a perception that the needs of the more charismatic species will unfairly trump those of the prey species. In our experience, genuine conservation conflicts often result in management inaction in the absence of a framework in which to assess likely impacts. Such a framework need not be complex [16], but any quantitative information can help initiate a conflict-resolution process. Any process that aims to balance competing objectives will be case-specific, but life-history parameters of the target species will impose some constraints. It is faster to reduce takes of salmon than to increase salmon production, and it is faster to increase salmon production than promote population growth in killer whales. The efficacy of salmon habitat restoration actions can often be measured within a decade [69], whereas similar measurements will take decades in studies of long-lived species like killer whales [29], [30]. This mismatch has implications for adaptive management strategies [72]. In the face of conflict, it is sensible to predict how prey will respond to management action, and monitor to ensure that management actions are achieving the desired effect. For most predator populations (i.e., all those whose population size is estimated rather than counted), the time delay will be exacerbated by low statistical power to detect whether management actions are working [73]. It is precautionary to order management actions to give priority to those that can halt population declines as quickly as possible, and the life-history attributes dictate that this will usually involve an initial focus on prey species. To inform decision-making in such cases, we see value in building models to predict likely ecosystem responses to management action, whether those models are qualitative [74] or quantitative ones [16]. When tradeoffs have to be made, we see two choices. One is to prioritise the species whose extinction probability is higher. The other is to construct management frameworks that acknowledge the fact that we can detect responses of populations of short-lived animals more quickly than those of long-lived species. Both of these choices are difficult, imperfect, and reflect societal values as much as scientific questions. In that light, it is important to be as transparent as possible about the tradeoffs and logistical constraints, and to be specific about what the management actions aim to accomplish.

## Supporting Information

Text S1 **This supporting information file includes expanded descriptions of datasets used in the analyses, full details of the modeling approach, and additional summary statistics and model outputs.**
(DOCX)

Table S1 **Mean caloric value of prey items fed to killer whales at SeaWorld.**
(DOC)

Table S2 **Three growth model outputs compared for length at age data from captive records.**
(DOC)

Table S3 **Parameters of the fixed effects in the energy versus length model.**
(DOC)

Table S4 **Results of the Tukey's post-hoc tests comparing across reproductive classes.**
(DOC)

Table S5 **Killer whale body lengths from datasets used in these analyses in comparison to those reported in previous studies.** Our best estimate of asymptotic body length (cm) of southern resident killer whales (SRKWs) is shown in **bold**, and is based on the 80^th^ percentile of the distribution of body lengths of killer whales taken from the North Pacific in the IWC catch records.
(DOC)
