# Supplementary material for: Competing Conservation Objectives for Predators and Prey: Estimating Killer Whale Prey Requirements for Chinook Salmon
Source: PLoS One. 2011 Nov 9;6(11):e26738. doi: 10.1371/journal.pone.0026738 (PMC3212518; doi:10.1371/journal.pone.0026738)
Supplement: Text S1 — This supporting information file includes expanded descriptions of datasets used in the analyses, full details of the modeling approach, and additional summary statistics and model outputs. (DOCX) [file pone.0026738.s001.docx]

**Supporting Information**

*Mass at length from live-capture records and data from captive animals*

Prey requirements vary as a function of body mass, but length data are more commonly available for whales than mass records. To estimate mass at length, we fit a power function (Equation 1) to data obtained from wild killer whales caught in a live-capture fishery [43], using ecotype as a candidate covariate (see details below). The live-capture length and weight data from Hoyt [43] included records from four different killer whale ecotypes; Icelandic (n=11), northern resident (n=15), southern resident (n=18) and North Pacific transient (n=4). The resulting mass-at-length curve was compared to a widely-used curve for the species that was reported before the various ecotypes were described [75].

$W=aL^{b}$, where W-weight (kg), L-length (cm) {1}

Parameters *a* and *b* were estimated using a linear regression model (Equation 2). Then models were compared with both fixed and random effects for *a* and *b* for each ecotype

$\ln(W)=\ln(a)+b(\ln(L))$ {2}

The model was then fit to morphometric data collected from killer whales held at three SeaWorld facilities in the US [76], with a random effect on the intercept and slope for individual whales to account for repeated measures [42].

*Length at age for captive killer whales*

For free-ranging SRKWs, which have been studied extensively for 30 years, age estimates are available for every animal in the population, but not lengths. Body length at age was modelled from records for 30 captive killer whales (29 of Icelandic origin, and one adult female northeast Pacific resident). The captive killer whale data set included nearly 3,000 records for length, age, mass and food consumption for 30 whales (13 males and 17 females). Following Winship et al. [41], three standard growth models were fitted and compared: von Bertalanffy (Equation 3), Gompertz (Equation 4) and a logistic growth function (Equation 5). Each model has an initial length at age 0 (*b* for equations 3 and 4, *a*⋅(1+*b*)^-1^ for equation 5), a growth rate, *c*, and an asymptotic length, *a*. Each model had the following three assumptions: males and females have same length at birth; males and females may differ in rate of growth and in asymptotic length; and all models enforced positivity in the growth rate parameter by taking the exponent, therefore the random effect is log normal. AIC was used for model selection. A nonlinear mixed-effects model was fitted in R package nlme (R core Development Team 2009) to estimate growth parameters for captive killer whales, while accounting for repeated measures within individuals, to allow testing for differences in growth rate, *c*, and asymptotic growth, *a*, between males and females.

von Bertalanffy: $L=a+(b-a)\exp\left[-cA\right]$ {3}

Gompertz: $L=a\cdot\exp\left[-b\cdot\text{e}^{-cA}\right]$ {4}

Logistic: $L=\frac{a}{1+b\cdot\exp\left[-cA\right]}$ {5}

*Estimating energetic value of prey consumed (kilocalories per day)*

Feeding records consisted of killer whale food consumption rates (kg prey per day for each species, averaged over each calendar month) and the average energy density of representative prey samples (kcal/kg wet weight, determined via bomb calorimetry or extrapolated from composite composition analyses; Table S1). The number and frequency of caloric content analyses were not consistent among prey types, therefore a mean energy density value was used for each prey type. The product of the average prey energy density (kcal/kg wet weight) and the average daily food intake (kg/day) per month for each individual prey type was summed across species to yield average daily energy intake levels for each whale (kcal/day) for each month.

Daily energy intake was modeled separately for males and females. Females were further placed into one of four categories describing reproductive status: (1) ‘Single’ (neither pregnant, lactating nor immediately post-lactation); (2) ‘Pregnant’ (inferred from blood hormone levels); (3) ‘Lactating’ (recorded until month 3 post-birth); and (4) ‘Post-lactation’ (six months after lactation records end to allow for possibility of continued partial lactation and a recovery period). In the wild, lactation may extend to at least 2 years [77], and may not be as abrupt as was treated in the captive records and these analyses, but this categorical approach is a useful starting point that allows for straightforward model interpretation.

*Energetics modelling of prey consumption as a function of killer whale body size*

Mean daily energy consumption was modeled as a function of body length, rather than mass or age, because this is more commonly available than age or mass data for cetaceans [78]. New photogrammetric and laser-grammetric methods allow length to be measured in the field [61].

A non-linear mixed-effects model was used to model daily energy consumption at a given length for males using the R package nlme. For males, length was treated as a covariate and a random effect on individual accounted for repeated measures. For females, a non-linear mixed-effects model was also used with length as a covariate and reproductive status as a factor using the R package nlme. The energy-versus-length model was given by

$\ln(E)=\ln(a)+b(\ln(L)$ {6}

where *E* is the energy consumed per day in Kcal, *L*, is the length of the whale in cm, and a random effect for individual is included on the intercept, *b*. Initially, no difference in daily energy consumption at length was assumed between males and females. Exploratory analyses suggested that males and females shared a common shape of the curve (exponent), but different intercepts. Intercepts were compared for females according to the four (previously described) categories of reproductive status. Tukey’s multiple post-hoc comparisons were used to determine which pair-wise comparisons of reproductive status differed significantly.

*Body size of free-ranging killer whales*

Whaling records (provided by Cherry Allison, International Whaling Commission, The Red House, 135 Station Road, Impington, Cambridge, Cambridgeshire CB24 9NP, UK) were used to assess the extent to which body sizes of captive Icelandic killer whales (on which our analyses were based) were comparable to those of killer whales harvested in the northeast Pacific. Body weights could not easily be compared, because commercially caught killer whales were rarely weighed, but length measurements of Icelandic whales were compared with those from >3,000 killer whales taken worldwide.

*Estimating length, weight and energy requirements of free-ranging SRKWs*

Parameter estimates from models describing length at age, mass at length and daily energy consumption were then applied to data available for the southern resident killer whale (SRKW) population (courtesy Ken Balcomb, Center for Whale Research, PO Box 1577, Friday Harbor WA 98250 USA). Demographic data (age, sex, reproductive status) from CWR’s census of the population conducted annually since the 1970s were used to construct an age distribution of the SRKW population in 2009. The energetics and growth models were used to predict length, mass, daily and annual energy requirements for SRKWs of different age-sex classes (males; females with calves (‘post-lactation females’), females without calves (‘single’ females); lactating females; and juveniles). Pregnant females were not considered separately because it is difficult to determine pregnancy status in wild whales and there was no significant difference in energy demands of pregnant and non-pregnant, non-lactating females. Calves <1 year were assumed to obtain all nutrient requirements through their mothers’ milk. These estimates were summed to estimate daily energy requirements for the population, and scaled up to an annual requirement. For illustrative purposes, this caloric requirement was converted to the number of fish needed to support the population if they fed solely on Chinook salmon (i.e., the “100% scenario”). We first considered a value of a Chinook salmon of 16,386 kcal/fish, which was reported from samples taken from the SRKW core habitat in summer [34]. Next, we estimated the caloric value of a typical prey item as follows: preferred prey was a 4-year-old Chinook of fork length 80.8 cm and mass 8.5 kg [17]; caloric content of Chinook is 2,200 kcal/kg [44, p. 57] or 1,279 kcal/kg (converted from 5.35 kJ/g in [45]); therefore each Chinook was estimated to contain 18,700 kcal (“calorie-rich” scenario) or 10,869 kcal/fish (“lean” scenario). Note that the values we used for preferred prey are larger than average fish in the wild, due to strong evidence for SRKW prey selectivity toward larger fish [17]. Finally, we estimated a more realistic number (i.e., the “summer scenario”) of Chinook salmon consumed by SRKWs based on the proportion of Chinook in the whales’ summer diet (May -September), which has been studied most extensively [18].

**Results**

*Mass at length from live-capture records*

There was no support from the live-capture data for differences among four ecotypes (Figure 1) in growth rate (ANOVA, P=0.2526) or asymptotic length (ANOVA, P=0.2575). Similarly, there was no statistical support for a difference between males and females in growth rate (ANOVA, P=0.4306) or asymptotic length (ANOVA, P=0.4184).

There was strong support from the data (∆AIC=9.37) for excluding random effects on growth rate, asymptotic length or ecotype, therefore data were pooled across ecotypes. We estimated that *a* = 6.7e-05 (95% CI: 1.7e-05 to 2.6e-04) and *b* = 2.8 (95% CI: 2.5 to 3.0). The curves of mass at length for each of the four ecotypes are shown in Figure 1.

Models of mass at length for captive animals were identical to those for the live-capture data, except that the former included a random effect for individual on either the slope or intercept due to the repeated measures structure of the captive data (a model with random effects on both the intercept and slope failed to converge). The two models had similar likelihood values, and parameter values were very similar for the two parameters: i.e., random effect on intercept or slope were the same at this level of significant digits: *a* = -9.4 (-9.2 to -9.6), *b*=2.73 (2.69 to 2.76).

*Length at age for captive killer whales from SeaWorld records*

We excluded data from the single North Pacific killer whale (an adult female from the northern resident population) because this individual was substantially longer than Icelandic whales. The resulting data set included 29 captive records, representing 13 males and 16 females, all of Icelandic origin.

The Gompertz growth model was best supported by the length-at-age data from captive killer whales from SeaWorld (∆AIC=34.23; Table S2). The Gompertz model was used to test for sex differences in growth rate and asymptotic length parameters (*a* and *c*). A model with fixed effects by sex was compared to a model in which growth parameters were identical between males and females. Only weak statistical evidence was found to support differences in growth parameters between males and females (∆AIC=1.08; log-likelihood ratio test P=0.079). Biologically, we know that males reach a larger size than females [48,49], so either we have too few data to detect this, too few time series, or both. Therefore, we proceeded on biological grounds, assuming males and females could differ in both asymptotic length and growth rate. We therefore fit a nonlinear mixed-effects model to the captive data to estimate growth parameters and length parameters for males and females using sex as a fixed factor, as well as random effects on these parameters distributed among individual animals to account for repeated measures. That is, the model was *L* = *a* ⋅ exp[b ⋅ exp( c ⋅ Age)], where *L* is body length and *a, b,* and *c* are parameters to be estimated. To enforce positivity in the parameter values, we estimated *a* = exp(*a_1_*)*, b* = exp(*b_2_*), and *c* = exp(*c_2_*) and so random effects were log-normally distributed. Estimated parameter values were *a*_1,female_ = 558.7, *a*_1,male_ = 629.1, *b*_1_ = -0.22, *c*_1,female_ = -1.3, and *c*_1,male_ = -1.7, with standard deviations for random effects 84.8 on *a*_1_ and 0.58 on *c*_1_.

*Energetics modelling*

Parameters of the fixed effects in the energy versus length model and 95% confidence intervals are shown in Table S3. There was strong support for a model in which the intercept varied according to reproductive class (∆AIC=46.92; log-likelihood ratio test P<0.001; Tukey’s post-hoc tests are given in Table S4). By way of example, for a 32-year-old female, parameter estimates in Table S3 indicate that prey intake during lactation were 42% higher than for a same-aged female that was not pregnant or nursing.

*Maximum body size of free-ranging killer whales inferred from whaling records*

While there was no evidence from the live-capture fishery data for variation in mass at length across ecotypes (Figure 1), there was strong evidence of global variability in maximum body length in the catch records (International Whaling Commission, Table S5). Live-capture fisheries tend to target juveniles [79], so whaling records should provide a more complete sample to determine asymptotic length than live-capture fishery data. The largest killer whales killed in the northeast Pacific attained greater body length than those taken in any of the live-capture fisheries, and the largest whales in whaling records from both the North Atlantic and North Pacific attained greater length than the captive Icelandic whales for which prey consumption data are available (Table S5). In fact, recall that the single captive northern resident killer whale was treated as a statistical outlier in the growth equations. Therefore, any inference from captive whales to free-ranging killer whales from the northeast Pacific involves some extrapolation.

*Estimated prey requirements of SRKWs from energetics models and body size predictions*

If SRKWs were as small as captive Icelandic killer whales and exclusively consumed Chinook, we estimate that the SRKW population would need the energetic equivalent of approximately 171,000 large Chinook salmon annually (Table 1). If we used the maximum body size found in IWC whaling records for killer whales taken from the North Pacific, we would estimate that the SRKW population required the energetic equivalent of approximately 397,000 fish (Table 1). We propose the 80^th^ percentile of the length distribution in North Pacific whaling records as a best estimate of asymptotic body length, as follows (Table 1, **bold**). Killer whales grow to a certain age and then stop growing when they reach physical maturity at approximately 20 years of age [75], thus all killer whales older than 20 have reached maximum individual size. The asymptotic maximum size is the mean of all whales that have reached physical maturity, and relating this to an observed length distribution is therefore dependent on population-specific estimates of survival. In the SRKW demographic data (Center for Whale Research), 35 of 87 (i.e., 40%) individuals were ≥ 20 years of age. Therefore, asymptotic length should be about the 80th percentile in the SRKW population, assuming that half of the animals are above this length and half below. If we used the 80^th^ percentile as the estimate of asymptotic length, the population-level requirement is the energetic equivalent of approximately 241,500 large Chinook salmon annually (Table 1). These simplistic conversions are illustrative, because the diet of SRKW also includes fish other than Chinook salmon and because the winter diet is poorly studied [17,18,29]. Assuming a diet of 83% Chinook, from May-September [18], the minimum estimate of population-level requirement of Chinook salmon, i.e. only during summer months is approximately 83,500 [5/12*0.83*241,507] (shown in columns labelled “Summer” in Tables 1 and 2).

References

75. Bigg MA, Wolman AA (1975) Live-capture killer whale (*Orcinus orca*) fishery, British Columbia and Washington, 1962-73. J Fish Res Bd Can 32: 1213-1221.

76. Clark ST, Odell DK, Lacinak CT (2000) Aspects of growth in captive killer whales (*Orcinus orca*). Mar Mamm Sci 16: 110-123.

77. Ford JKB, Ellis GM, Balcomb KC (2000) Killer whales: The natural history and genealogy of *Orcinus orca* in British Columbia and Washington State. Vancouver: University of British Columbia Press. 108 p.

78. Trites AW, Pauly D (1998) Estimating mean body masses of marine mammals from maximum body lengths. Can J Zool 76: 886-896.

79. Williams R, Lusseau D (2006) A killer whale social network is vulnerable to targeted removals. Biol Letters 2: 497-500.

80. Rosen DAS, Trites AW (1999) Metabolic effects of low-energy diet on Steller sea lions, *Eumetopias jubatus*. Physiol Biochem Zool 72: 723-731.

81. Olesiuk PF (1993) Annual prey consumption by harbor seals (*Phoca vitulina*) in the Strait of Georgia, British Columbia. Fish Bull 91: 491-515.

82. Klinowska M (1991) Dolphins, porpoises and whales of the world: the IUCN Red Data Book: IUCN.
