# Supplementary material for: Competing Conservation Objectives for Predators and Prey: Estimating Killer Whale Prey Requirements for Chinook Salmon
Source: PLoS One. 2011 Nov 9;6(11):e26738. doi: 10.1371/journal.pone.0026738 (PMC3212518; doi:10.1371/journal.pone.0026738)
Supplement: Table S1 — Mean caloric value of prey items fed to killer whales at SeaWorld. (DOC) [file pone.0026738.s002.doc]

**Table S1.** Mean caloric value of prey items fed to killer whales at SeaWorld

| **Prey item** | **Average caloric content**  **(kcal/kg wet weight ± SD)** | **Sample size (number of 0.91 kg sub-samples of prey analyzed via bomb calorimetry)** |
| --- | --- | --- |
| Capelin | 1207.1±33.1 | 34 |
| Herring | 1535.3±30.7 | 41 |
| Mackerel | 1205.8±22.1 | 16 |
| Salmon | 890.0±0.0 | 4 |
| Sardine | 1510.8±32.3 | 21 |
| Smelt | 1367.1±30.9 | 7 |
| surf smelt | 1100.0±32.7 | 2 |
| white bait | 1103.3±50.0 | 3 |
| hake^1^ | 1170.0 | NA |
| squid^2^ | 620.0 | NA |

^1^ Composition data not reported by SeaWorld, so a value from similar analyses performed at the Vancouver Aquarium was used [80].

^2^ Composition data were not reported by SeaWorld, so a value was used from [81].
