# Supplementary material for: Competing Conservation Objectives for Predators and Prey: Estimating Killer Whale Prey Requirements for Chinook Salmon
Source: PLoS One. 2011 Nov 9;6(11):e26738. doi: 10.1371/journal.pone.0026738 (PMC3212518; doi:10.1371/journal.pone.0026738)
Supplement: Table S2 — Three growth model outputs compared for length at age data from captive records. (DOC) [file pone.0026738.s003.doc]

**Table S2:** Three growth model outputs compared for length at age data from captive records.

| **Model** | Df | AIC | BIC | logLik |
| --- | --- | --- | --- | --- |
| von Bertalanffy | 9 | 15615.48 | 15666.30 | -7798.741 |
| Gompertz | 9 | 15247.51 | 15298.33 | -7614.756 |
| Logistic | 9 | 15281.74 | 15332.56 | -7631.871 |
