# Supplementary material for: Competing Conservation Objectives for Predators and Prey: Estimating Killer Whale Prey Requirements for Chinook Salmon
Source: PLoS One. 2011 Nov 9;6(11):e26738. doi: 10.1371/journal.pone.0026738 (PMC3212518; doi:10.1371/journal.pone.0026738)
Supplement: Table S3 — Parameters of the fixed effects in the energy versus length model. (DOC) [file pone.0026738.s004.doc]

**Table S3:** Parameters of the fixed effects in the energy versus length model

lower estimate upper

*b* (Log length) 2.504581 2.637310 2.770040

ln(*a*) lactating -5.756381 -4.924433 -4.092485

ln(*a*) Male -6.020916 -5.163996 -4.307075

ln(*a*) post-reproductive -5.945881 -5.115386 -4.284891

ln(*a*) pregnant -6.166349 -5.341221 -4.516093

ln(*a*) Single female -6.096136 -5.275155 -4.454173
