# Supplementary material for: Competing Conservation Objectives for Predators and Prey: Estimating Killer Whale Prey Requirements for Chinook Salmon
Source: PLoS One. 2011 Nov 9;6(11):e26738. doi: 10.1371/journal.pone.0026738 (PMC3212518; doi:10.1371/journal.pone.0026738)
Supplement: Table S4 — Results of the Tukey's post-hoc tests comparing across reproductive classes. (DOC) [file pone.0026738.s005.doc]

**Table S4:** Results of the Tukey’s post-hoc tests comparing across reproductive classes

Estimate Std. Error z value Pr(>|z|)

Male vs lactating == 0 -0.23956 0.09116 -2.628 0.05748

post vs lactating == 0 -0.19095 0.07491 -2.549 0.07038

pregnant vs lactating == 0 -0.41679 0.06425 -6.487 < 0.001

Single vs lactating == 0 -0.35072 0.06194 -5.663 < 0.001

post vs Male == 0 0.04861 0.08309 0.585 0.97406

pregnant vs Male == 0 -0.17722 0.07330 -2.418 0.09787

Single vs Male == 0 -0.11116 0.06841 -1.625 0.45084

pregnant vs post == 0 -0.22583 0.05209 -4.336 < 0.001

Single vs post == 0 -0.15977 0.04928 -3.242 0.00915

Single vs pregnant == 0 0.06607 0.03000 2.202 0.16006
