# Supplementary material for: Competing Conservation Objectives for Predators and Prey: Estimating Killer Whale Prey Requirements for Chinook Salmon
Source: PLoS One. 2011 Nov 9;6(11):e26738. doi: 10.1371/journal.pone.0026738 (PMC3212518; doi:10.1371/journal.pone.0026738)
Supplement: Table S5 — Killer whale body lengths from datasets used in these analyses in comparison to those reported in previous studies. Our best estimate of asymptotic body length (cm) of southern resident killer whales (SRKWs) is shown in bold, and is based on the 80th percentile of the distribution of body lengths of killer whales taken from the North Pacific in the IWC catch records. (DOC) [file pone.0026738.s006.doc]

**Table S5:** Killer whale body lengths from datasets used in these analyses in comparison to those reported in previous studies. Our best estimate of asymptotic body length (cm) of southern resident killer whales (SRKWs) is shown in **bold**, and is based on the 80^th^ percentile of the distribution of body lengths of killer whales taken from the North Pacific in the IWC catch records.

| **Source** | **Sex** | **Area** | **Maximum** | **80th** | **95^th^** | **99^th^** | **N** |
| --- | --- | --- | --- | --- | --- | --- | --- |
| Hoyt [43]  Live Capture | F | Icelandic | 525 | 468 | 513 | 523 | 9 |
| Hoyt [43]  Live Capture | M | Icelandic | 455 | 436 | 453 | 454 | 6 |
| Hoyt [43]  Live Capture | F | NE Pacific Residents | 610 | 578 | 594 | 607 | 15 |
| Hoyt [43]  Live Capture | M | NE Pacific Residents | 700 | 642 | 687 | 697 | 19 |
| Hoyt [43]  Live Capture | F | NE Pacific Transient | 427 | 421 | 425 | 427 | 3 |
| Hoyt [43]  Live Capture | M | NE Pacific Transient | 467 | 459 | 465 | 467 | 2 |
| IWC | F | Indian Ocean | 853 | 750 | 820 | 847 | 13 |
| IWC | M | Indian Ocean | 884 | 792 | 792 | 863 | 25 |
| IWC | F | North Atlantic | 762 | 640 | 716 | 753 | 16 |
| IWC | M | North Atlantic | 914 | 731 | 799 | 889 | 29 |
| IWC | F | North Pacific | 780 | **630** | 710 | 742 | 141 |
| IWC | M | North Pacific | 820 | **700** | 770 | 804 | 260 |
| IWC | F | Norway | 853 | 610 | 671 | 752 | 933 |
| IWC | M | Norway | 975 | 701 | 762 | 853 | 1462 |
| IWC | F | Southern Ocean | 770 | 660 | 690 | 740 | 599 |
| IWC | M | Southern Ocean | 900 | 770 | 850 | 890 | 661 |
| Klinowska [82] | F | Global | 850 | - | - | - | - |
| Klinowska [82] | M | Global | 980 | - | - | - | - |
| SeaWorld | F | Icelandic | 626 | 560 | 598 | 620 | 16 |
| SeaWorld | M | Icelandic | 685 | 604 | 651 | 678 | 12 |
| Bigg and Wolman [75] | F | NE Pacific | 625 | - | - | - | 27 |
| Bigg and Wolman [75] | M | NE Pacific | 698 | - | - | - | 29 |
